# The First Foods Qualitative Study: Using the Developmental Niche Framework to Understand Caregiver and Infant Feeding Interactions During the Complementary Feeding Period

**DOI:** 10.3390/nu18071121

**Published:** 2026-03-31

**Authors:** Susan L. Johnson, Katherine J. Barrett, Kameron J. Moding, Catherine A. Forestell

**Affiliations:** 1 Section of Nutrition, Department of Pediatrics, Anschutz Medical Campus, University of Colorado Denver, Mail Stop #C225, 12631 E. 17th Ave, Aurora, CO 80045, USA; katiebarrett23@gmail.com; 2 Department of Human Development and Family Science, Purdue University, 1200 Mitch Daniels Blvd., West Lafayette, IN 47907, USA; kmoding@purdue.edu; 3 Department of Psychological Sciences, William & Mary, 540 Landrum Drive, Williamsburg, VA 23185, USA; caforestell@wm.edu

**Keywords:** complementary feeding, infants, Developmental Niche, child-feeding

## Abstract

**Objectives**: The transition to complementary feeding represents an important interval in child nutrition and development. Nutrient demands for growth are high, yet less is known regarding how caregivers make decisions regarding the introduction of solid foods to their infants and what influences their choices and feeding practices. **Methods**: Semi-structured interviews were conducted via Zoom with caregivers (N = 46, 83% mothers) of typically developing children (6–24 months of age) residing in the United States. A content analytic approach, with consensus coding performed by team members, was undertaken. The Developmental Niche framework guided thematic analysis. **Results**: Four major themes and four subthemes were identified: (1) *Caregivers’ Approach Introducing Solid Foods with Anticipation and Concern*, including subthemes of the (a) timing and order of complementary foods (CF) offered to children and (b) foods caregivers avoid offering; (2) *Caregivers’ and Children’s Learning*, including subthemes of (a) children’s rapid learning and skill development, and (b) the concurrent rapid demands for changes in food parenting; (3) *Drivers of Caregivers’ Decisions Related to Offering Solid Foods to their Children;* and (4) *The Goal of CF: Integration of the Child into Family Mealtimes*. **Conclusions**: Caregivers seek to provide adequate nutrition while balancing children’s health needs with the challenge of encouraging acceptance of family foods and respecting individual preferences. Juggling myriad demands (e.g., time, convenience, other family members, cultural traditions, and expectations), caregivers seek to help their children develop a healthy relationship with food.

## 1. Introduction

The complementary feeding (CF) period, defined here as approximately 6–24 months of age, represents an important developmental transition from a milk-based to a solids-based diet and culminates in the transition to family foods of the table [1,2]. In addition to the changing nutrition needs and eating goals of consuming family foods, this period also provides increasing opportunities for the child to participate in the social and communal aspects of eating, including shared family meals, celebrations, and holiday gatherings. Concomitantly, infants and toddlers experience rapid cognitive and gross and fine-motor development, which facilitates their ability to manipulate and consume a wide variety of foods [3,4]. Examples of motor skill development include increasing head and neck control, sitting stably and independently, oral motor development (e.g., lateralizing food in the mouth, so that they can chew and swallow solid foods), and developing palmar and then pincer grasps that enable self-feeding [3]. At the same time, children’s language and cognitive development flourish within the context of extensive social interactions, including those that take place at the family table [5,6]. For example, they learn to communicate hunger and satiety in new ways, such as learning new food-related signs to communicate when they want “more” (food) or are “all done”, bringing eating utensils to caregivers, pointing towards other family members’ plates, and saying or shaking their head “no” [7].

Several studies conducted in the United States and in other parts of the world have examined what motivates caregivers’ decisions to employ particular CF practices [8,9,10,11,12], including the timing of the introduction of complementary foods and what kinds of foods are offered [13,14]. Although fewer studies in the United States have focused on the underlying cultural and family belief systems that influence caregivers’ CF decisions, studies in other settings have demonstrated that attitudes and expectations vary among and within different cultural settings. These studies most often emphasize the environmental influences on children’s food acceptance rather than exploring how their eating develops over time or how their appetitive traits influence caregiver feeding strategies. For example, Schwartz et al. (2013) reported that French mothers consider it important to expose their children to a diverse range of foods during complementary feeding to “educate the palate” to develop a pleasure for a variety of healthy foods [15]. Norlyk et al. (2019) highlighted parents’ lived experiences during CF [16]. They reported that Danish parents felt anxious, pressured, and unprepared in implementing responsive feeding during CF due to notions regarding “perfect parenting” and difficulty interpreting their infants’ reactions to food, including fears of choking and infants’ distaste and food rejection. However, the bidirectional nature of parent–child interactions, the impacts of culture on parent beliefs and practices, and the way the child simultaneously influences the environment in which they are developing (e.g., children’s responses to foods sometimes shaping whether foods are repeatedly offered) have not often been considered holistically.

Thus, children’s eating development occurs within the context of various environments and social interactions, with caregivers’ expectations for children’s eating behaviors evolving in parallel with children’s developmental progression during the CF period. The Developmental Niche provides a theoretical framework for examining the bidirectional nature of how children develop within an environment, or “niche,” and how the child and their niche mutually adapt and progress through developmental stages [17]. The Developmental Niche is a multidimensional system composed of (1) the child’s physical and social setting, (2) culturally influenced norms of child care and rearing, and (3) caregivers’ belief systems and ethnotheories regarding child development, parenting, and family habits and routines, each of which informs their decision-making. An example of a physical aspect of the eating setting might be whether adequate food and nutrient sources are available for the child’s development and growth. Cultural norms of child-feeding and foods considered appropriate for infants during complementary feeding influence caregivers’ feeding decisions and can be based upon organizational or best practice guidelines. Lastly, caregivers’ perceptions of their children’s nutrition and developmental needs, their beliefs about effective food parenting, and how food-related customs are integral to mealtimes, holidays, and celebrations exemplify how caregiver belief systems contribute to the niche. These three components are conceived to operate as a system, each adapting to the other; according to the bidirectional nature of the theory, the child also influences each component.

The Developmental Niche has been a guiding framework for many different areas of inquiry, including learning and play contexts for young children [18], language development [19], children’s success in school settings [20], and the emergence of maladaptive behaviors [21]. Most relevant to the development of children’s eating behaviors are those focused on families with preschoolers, examining parents’ ethnotheories of food parenting and their values, beliefs, and motivations for feeding practices, which can shape young children’s eating patterns [22].

The present study sought to understand and describe processes and conceptualizations of CF (i.e., younger children who are infants and toddlers) among caregivers living in the United States, including how caregivers’ understanding of child development and family contexts influence what, when, and how they introduce complementary foods to their infants. Consistent with Developmental Niche theory, we queried caregivers regarding beliefs and attitudes that underlie their decisions about (1) when and which solid foods they offer their children and how these relate to children’s nutrition needs and preferences; (2) their expectations for children’s eating development and how their child’s stage of development influenced their decisions; and (3) what kinds of external influences impact their decisional processes related to CF. We sought to investigate whether and how each of these factors interact with one another and endeavored to identify the ways they collectively shape children’s eating development. Finally, we aimed to explore how their children’s development and unique characteristics, in turn, influenced caregivers’ beliefs, attitudes, and feeding practices.

## 2. Methods

### 2.1. Recruitment and Data Collection

Data were collected during May and June of 2022 as part of a larger multiple methods parent study, the *1st Foods Study*, the primary aim of which was to investigate how caregivers in the United States decide when, whether, and how to offer meat to their children during the CF period (6–24 months). Participants for this qualitative study were recruited through Qualtrics (Provo, UT, USA), advertisements on social media, and snowball sampling. Qualitative interviews were chosen as a method to gather individual, in-depth perspectives on the social and personal beliefs about complementary feeding and to allow each individual to relate their experiences and perceptions [23]. Participants completed a brief online screening survey which asked about demographic information, including caregiver and child race and ethnicity, caregiver marital status, education, and employment, the number of children in the household, whether the household was of limited income (defined as $51,000 or less, or ≤185% of the 2022 U.S. Federal Poverty Level for a family of four; [24]), and whether the household experienced food insecurity over the past 12 months based on a 2-item screener [25,26]. Caregivers were eligible to participate if they were a primary caregiver for a child between 6 and 24 months of age who was receiving solid foods, if their child was ≥37 weeks gestation at birth and did not have food allergies or a diagnosed medical condition that might affect eating. In total, 46 caregivers living in the United States were contacted by a member of the research team, scheduled for interview, and interviewed for the present study.

Semi-structured, in-depth interviews (see Table 1) were conducted via Zoom (Zoom Video Communications Inc., San Jose, CA, USA) by trained members of the research team using a shared interview guide [27]. Questions were developed by the research team and organized into 4 categories: the factors related to the timing, process, and decisions related to introducing complementary foods to infants; animal source foods, specifically as complementary and family foods (analyzed separately and focused on in a separate manuscript); nutrition literacy related to feeding infants and children; and information sources for CF. Questions were piloted in a mini-focus group (4 participants) held via Zoom to clarify the wording and intent of questions with participants who were similar to our target group. Subsequently, the decision was made to add screening questions before the interview questions to verify the age and sex of the child, confirm the introduction of solid foods, verify caregiver demographics (self-described gender and race/ethnicity), and confirm the number of children in the household. The procedure was explained to each participant, and verbal consent to video-record interviews on Zoom was obtained from each participant. Interviewers took field notes during each interview, which were shared with the research team. The research team met intermittently throughout data collection to discuss what they were learning from caregivers and to determine when saturation was achieved. Interviews subsequently were transcribed by a Health Insurance Portability and Accountability Act of 1996 (HIPAA)-compliant vendor (Datagain Transcript Services, Secaucus, NJ, USA). Transcripts were verified by a member of the research team, de-identified, and uploaded into NVivo (NVivo20, QSR International, Doncaster, Australia) for organization and coding.

This study was conducted in accordance with the Declaration of Helsinki and determined to be exempt by the Colorado Multiple Institutional Review Board (Protocol #22-0015, approved 22 February 2022). Participants were notified of the study’s procedures in writing. Interviewers reviewed the study’s procedures with the participants, provided the opportunity to ask questions, and subsequently obtained verbal consent at the beginning of each interview. Participants were given a gift card with a value of $30 US.

### 2.2. Analysis

The focus of the present analysis relates to the first category of interview questions: the introduction of complementary foods. The analysis was guided by the research question: what factors, such as caregiver experiences and beliefs, influence decisions regarding the timing and methods of introducing complementary foods to infants? A conventional content analytic approach was employed to achieve a descriptive understanding of caregivers’ conceptions of CF [28]. The initial codebook was developed by the research team based on the content of the questions, interviewer notes, and team discussions. A team of 4 coders was trained by engaging in conversations about reflexivity, reviewing the codebook and interview notes, engaging in team coding with a consensus coding process, and then engaging in individual coding with weekly team meetings to resolve coding differences [29]. The coding team revised the codebook as needed to improve clarity. Once coding was completed, members of the research team met to conduct thematic analysis on the exported coded text segments [30]. Notes were documented and shared with the entire research team, who met on multiple occasions to discuss interpretations of the ongoing analysis.

The analysis and interpretation of the data were shaped by each of the analysts’ own perspectives. One team member is an early childhood nutritionist who identifies as White and middle-class and has a clinical and research perspective that she uses when interpreting qualitative data. The second member is a biocultural anthropologist who identifies as White and middle-class and has a young child with her partner. Her perspective has been shaped by her embeddedness within the White culture in the United States as well as by her engagement with infant feeding research conducted internationally. The third is a developmental scientist who identifies as White and middle-class. Her perspective was further shaped by her experiences conducting research with parents and their young children. The last team member identifies as White and middle-class and is a quantitative experimental psychologist who studies the development of flavor preferences in young children. These identities and practices shaped the authors’ interactions with participants during the interview process and informed their engagement with, and interpretation of, the data during thematic analysis.

## 3. Results

As shown in Table 2, most participants (N = 46) were mothers (83%), White (65%), married or partnered (89%), had high levels of education (71% had a 4-year degree or higher), were employed (74%), and had more than one child in the household (83%). Nearly one-quarter of participants came from households that were low-income or food-insecure (28% and 24%, respectively). A plurality of participating caregivers (41%) had infants (6–11 mo), 33% had young toddlers (12–17 mo), and 26% had older toddlers (18–24 mo). Half (50%) of children were female, and the majority (65%) were introduced to CF between 4 and 6 months of age.

**Figure 1:**
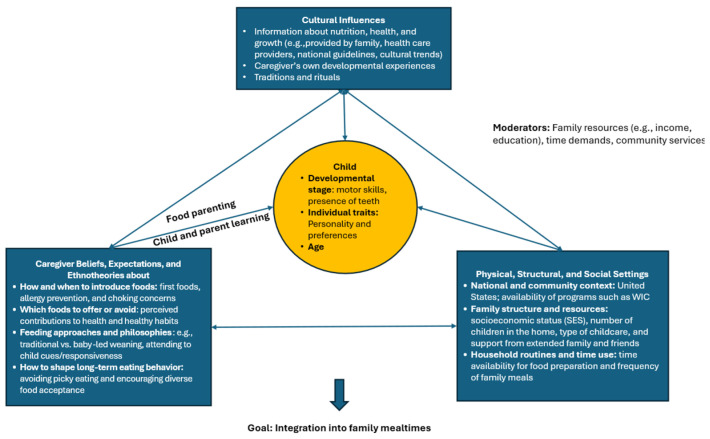
Developmental Niche Framework for Complementary Feeding. Caregiver complementary feeding practices through the lens of the Developmental Niche Framework (Harkness & Super, 1994; [19]). While a variety of drivers—such as culture, beliefs and expectations, structural settings, and the child’s individual characteristics—influence complementary feeding practices, caregivers’ goals focus on integrating their children into family mealtimes. Over time, parents’ food parenting strategies evolve alongside (or in response to) their children’s development, while children simultaneously acquire new skills through the food experiences provided by their parents.

Four major themes and four subthemes were identified that captured how caregivers thought about CF (Table 3). The first major theme was *Caregivers’ Approach Introducing Solid Foods with Anticipation and Concern*, including subthemes of the (1) timing and order of CF offered to children, and (2) foods caregivers avoid offering. The second major theme was *Caregivers’ and Children’s Learning,* including subthemes of (1) children’s rapid learning and skill development, and (2) concurrent rapid demands for changes in food parenting. The remaining themes included *Drivers of Caregivers’ Decisions Related to Offering Solid Foods to their Children* and *The Goal of CF: Integration of the Child into Family Mealtimes*. The relationships among these themes and subthemes, and their alignment with the Developmental Niche framework, are described below and are represented in Figure 1. Representative quotes have been included to illustrate each theme.

### 3.1. Caregivers’ Approach Introducing Solid Foods with Anticipation and Concern

Caregivers offered highly varied and detailed information regarding what, when, and how they approached offering CF to their children. Many looked forward to this transition, and their processes of offering CF were influenced by their beliefs, goals, and concerns about feeding. At other times, their choices were driven more by child-centered notions or observations of their child’s readiness for CF, and, in some cases, by circumstantial, social, or environmental considerations.

### 3.2. Timing and Order of CF Offered to Children

Most children were first introduced to solids around 6 months of age (range = 1.5–16 mo), and most often first foods were either cereal or fruit, followed rapidly by the introduction of vegetables. Foods from the meat group (including fish) were most often offered last, around 8–9 months of age, and with wide variation in timing (4–18 months).

The most common animal source foods offered first included eggs and yogurt (or other dairy); however, some caregivers reported offering some form of meat (including fish) first. When meat was offered, the first meat was most often chicken (offered at ~9 mo of age; range = 4–18 mo). The frequency of offering meat was most often reported to be several times per week, with about half of children in the 12–24-month age range being offered meat daily.

### 3.3. Foods Caregivers Avoid Offering

In general, caregivers reported being reluctant to offer food that they considered to be highly processed, high in sugar, too difficult for their child to consume at their current stage of development, or hazardous for children of this age. For example, if the ingredient list on labels of commercial infant foods was too long or complicated, some caregivers were skeptical of and eschewed these products. These caregivers more often “leaned in” to products or foods labeled as “organic” or that they prepared themselves at home. This was especially the case for infant/toddler meat-containing products, which were avoided because of perceptions of poor smell, texture, or taste. Foods perceived to contain too much sugar (and occasionally salt) were actively sidestepped to avoid “building bad habits,” to facilitate preferences for foods with less sweetness (like vegetables), and because they were perceived to be low in needed nutrients.

Predominantly, caregivers focused on safety when offering CF. The two concerns most often cited were choking and allergy, and for these reasons, many caregivers avoided offering meat, particularly red meat, because it was perceived to be a choking hazard and because of a concern that their child was too young to digest it properly. Dairy, and particularly cow’s milk, was avoided by some because of allergy concerns, though peanuts were often stated to be offered early and often by caregivers. Caregivers reported that they had been told to avoid honey, as it was not safe for young children.

### 3.4. Caregivers’ and Children’s Learning

During the CF period, caregivers reported that their children are learning skills in multiple areas (motor, sensory, exposure to food variety, and developing eating self-regulation) and are quickly gaining the ability to react to various tastes and foods and to express their liking (or dislike) for these foods. To engage in responsive feeding, caregivers focused on their young children’s cues in addition to offering different types and textures of foods to their children. They reported learning about the unique responses and characteristics of this child and navigating how to best attend to and support the child’s learning about food, eating, and their family culture. These reciprocal interactions between children and caregivers were described as both rewarding and challenging.

### 3.5. Children’s Rapid Learning and Skill Development

Although the specific foods and their nutrient content were considered by some to be an important aspect of the transition to a solids-based diet, many parents emphasized that CF is about much more than nutrition. Caregivers recognized that this interval is an important period of learning and a time of rapid skill development for young children, particularly motor skill development. Caregivers spoke of how infants and toddlers learn to manipulate foods with their mouths and hands. Some believed that opportunities for tactile exploration facilitated learning about food consistency and texture (beyond its taste and odor) and that sensory exploration helped to increase their child’s familiarity with the foods offered. Some caregivers aimed to provide sensory exploration opportunities, particularly by giving their children opportunities to self-feed; however, others preferred to diminish the amount of messiness involved in child feeding.

In short, many caregivers viewed the CF period as one in which children begin to develop eating as a life skill.

*I mean a lot of exposure, having the skills and… ugh [sigh], I think I’ve read somewhere too that the first, oh, I don’t know, the first two years, the first three years are the most nutritionally important for a child because it really sets up their palate, it sets up how their bodies are growing so rapidly, their brain is absorbing all those nutrients in that timeframe.*

Caregivers remarked that they wanted their children to learn to have “healthy relationships” with food, which included not viewing foods as “good” or “bad,” nor eating as an emotional coping mechanism. Caregivers favored supporting their children to learn to pay attention to their internal cues of hunger and satiety. Eating as a life skill also encompassed children learning to eat in different social settings and learning about culturally salient foods.

### 3.6. Rapid Changes in Food Parenting

Just as this was noted to be a period of rapid development for children, it was also a period of intense caregiver observation of their child’s abilities and needs to create timely opportunities for the child to learn healthy eating habits. Caregivers indicated a desire to provide a strong “foundation” of exposure to dietary variety in the hopes that their children will learn to eat “healthy” foods at older ages when they make more autonomous decisions about their dietary intake. Many hoped that setting this type of foundation would facilitate children’s learning and acceptance of a healthy and varied diet, reduce picky eating, and help their children fit into various social settings where food is consumed and plays a central role in the occasion as they got older.

Caregivers experienced a process of discovery when their child began to exhibit their own preferences, skills, and behaviors. Such learning and behaviors subsequently informed how caregivers proceeded with CF. For those who had other children, feeding experiences with their younger child were sometimes different than those with their older children. Caregivers used their observations to decide whether to continue feeding in a way that had worked for them previously or to attempt a new approach for CF that was more tailored to the current child.

As CF proceeded, children began to be able to communicate their preferences in new ways, and caregivers spoke of learning to navigate these new forms of communication. Caregivers looked for cues such as smiles, verbalizations, spitting out food, and throwing food on the floor to understand what their child was trying to communicate, and they tried to respond in ways they felt were appropriate. As infants took a more active role in their feeding, from verbally expressing their preferences to self-feeding, they began to behave more like the people around them.

*“We try and respect what she’s saying. If she’s saying all done or she doesn’t like it, I don’t wanna force her to eat something she doesn’t like.” (290)*

Several parents remarked that during this time, children began to imitate their family members, which was viewed as a welcome sign of maturation. Imitation was a signal that communication was occurring between two active participants.

Learning to navigate these new communications was one aspect of parenting that reflected parents’ changing role in feeding. Parents spoke of a continued responsibility to nourish their child, which brought many parents joy and satisfaction, and they found that their role in feeding expanded in new ways. For example, when children began to eat family foods, caregivers (and other family members) began to model the eating behaviors they wished their children might develop.

For some caregivers, the changing nature of their roles was met with some angst. Many felt a sense of responsibility for providing optimal nourishment for their children. Yet, when they felt circumstances demanded it (i.e., being time-constrained), some caregivers selected foods based on convenience or ease, such as providing fast food or ultra-processed food (e.g., chicken nuggets, puffs); they subsequently expressed feelings of self-blame or framed their actions as “lazy”. Caregivers also expressed concerns about the development of allergies, choking hazards, and the appropriateness of certain foods for children’s digestion. Although these concerns were not expressed exclusively by first-time parents, caregivers of multiple children tended to express more confidence about their choices. Several caregivers who had multiple children described being less concerned about which foods to offer and the timing of introduction and were more assertive in offering the foods they ate as a family earlier during CF.

### 3.7. Drivers of Caregivers’ Decisions Related to Offering Solid Foods to Their Children

Caregivers approached offering complementary foods with positive expectations and with the perspective that their child was ready to start eating solid foods. Sometimes a greater need for energy or specific nutrients for growth and health (e.g., protein and iron) drove the choice to start offering complementary foods to their child, and some mentioned being motivated by advice from their primary care provider. Additionally, many caregivers suggested that they followed their children’s cues, stating that they began offering foods when their young child showed interest in what the rest of the family was eating, or began reaching for foods when others were eating them. This was particularly the case when caregivers were preparing foods for other children and took the pragmatic approach of offering the infant or toddler what other children were already receiving.

*“But if I’m already going to make it for my daughter, then I’m just going to offer it to him at the same time as I am offering it to her.”*

The selection of which food to offer and the order of introduction of solids depended upon several factors. For many, ensuring that the first experience with solid foods was pleasant was of primary importance. Offering foods that were prepared to be easily ingested (i.e., soft) and digestible was important and was aligned with offering cereal or fruit first, and most often in a puréed or mashed form.

*“I don’t give him, uh, like bitter stuffs, I give him sweets stuffs, uh, he can enjoy. Uh, and I think he’s enjoying the process.”*

Others who endorsed a baby-led weaning approach offered foods that could be self-fed by the child (e.g., avocado and banana).

For those who were enrolled in the Supplemental Nutrition Program for Women, Infants, and Children (WIC), cost of food, and foods that were supported by WIC, influenced which foods were offered to their child (e.g., eggs). Caregivers reported feeling happiness and a sense of fulfillment when children progressed to consuming solids and had good experiences with incorporating new foods into their regimens.

As noted above, the introduction of animal-source foods generally came later in the CF period and seemed to be driven more by concerns of safety and developmental readiness. It was often stated that children needed animal-source foods to provide nutrients (protein in particular); however, concerns related to potential choking were given equal importance. The rationale to begin with eggs and/or yogurt was often framed in terms of children’s development—that children did not have (many) teeth. Further, those who were enrolled in WIC stated that eggs were a food that was supported by the program and this addressed budgetary concerns. However, fears about allergic reactions made other caregivers ambivalent about offering eggs and dairy, despite the awareness of guidance to offer nuts (another common allergen) early in the CF period.

*“So I wanted to kinda [wait to] see if we had an allergy for it,… uh-hmm think in terms of eggs, that was like a big—another one of those big, like, um, it’s a high allergy food.”*

Chicken and fish were more often chosen first because of their texture and “bland taste.” Additionally, ground meat, sometimes specially prepared for the young child and other times because the family was consuming it at as a meal, was considered appropriate and safe. Caregivers using a baby-led weaning approach stated that it was too “difficult to purée meat and we’re not doing that.” Thus, pieces were offered to their child to self-feed.

Caregivers also reported that their families’ cultures and their own upbringing related to food and eating influenced how they approached feeding their child. For example, many caregivers spoke of the extended family food culture; they wanted to honor their family traditions and food selections—particularly for holidays and celebrations. They spoke of offering their children the foods that were part of their culture but also related to their expectations that their child should learn to “eat what we eat” as a family. Sometimes these decisions deviated from how caregivers had been reared in that a few caregivers who had been raised in vegetarian families spoke of the importance of “making their own decisions” about what was appropriate for their child and their family. Furthermore, caregivers who leaned into more of a baby-led weaning approach related some pushback from extended family members whose concerns focused on safety and choking but remained determined to proceed on a course that they felt best for their child.

Some reported that their beliefs, goals and information related to nutrition and health drove the choices they made in offering foods during the complementary feeding period. In particular, caregivers seemed most focused on ensuring that their child was getting enough protein to support growth and development. Moreover, caregivers stated the importance of making sure that their child was exposed to a variety of tastes, textures and different foods with an eye towards warding off picky eating and helping their child develop a wide acceptance of foods—both to ensure adequate nutrition but also so that children would learn to accept foods that are important to the family culture. Lastly, caregivers were keen observers of their child’s developmental readiness and used their observations to determine the point at which to offer specific foods (e.g., those with more challenging textures) and the point at which their child could be offered the same foods that the rest of the family was eating at mealtime.

### 3.8. The Goal: Integration into Family Mealtimes

Caregivers acknowledged that introducing complementary and family foods into their child’s diet marks a significant transition for both the child and the family. Many aspects of feeding changed during this transition, including who fed the child, what children consumed, and where and how they ate. Many families embraced this opportunity to start integrating the child into the family’s social fabric during mealtimes. For many caregivers, this meant including the young child during mealtimes and often offering some version of family foods to the child while the whole family ate together. Even when caregivers’ meal schedules differed from those of their children, eating was often a family social event that included infants and toddlers.

Many parents recognized that the transition to consuming family foods was a process that would take time. Some were eager to encourage this transition as quickly as possible because it was easier and more convenient to prepare a single meal for the entire family. This sentiment was particularly prevalent for parents who had other children and were trying to meet the needs of other family members, although it was expressed by first-time parents as well. Preparing a single meal saved time and was perceived to be less of a hassle than providing separate meals for different family members.

As the foods children consumed changed during this transition, there were opportunities for family members to experience joy in this new stage of development.

*“Um, it was really cool seeing her eat what we eat and watching her get to try new foods and, you know, experience new things and get to eating with everybody else instead of bottles all day long.”*

Several caregivers expressed happiness in the recognition that their child’s participation in family meals made them seem more “grown up”. There was also shared joy found in the mutuality of the collective experience and in sharing foods that were enjoyed by both the caregiver and child, and often other family members as well. Several parents expressed that their young child took pleasure in sitting at the table with the family and eating foods in the same way they observed their family members eating. Thus, meeting the goal of including the young child in family meals brought a sense of satisfaction to caregivers and children alike.

## 4. Discussion

This study investigated caregiver decision-making during complementary feeding (CF) through the Developmental Niche framework, positioning CF as a dynamic developmental process rather than a series of isolated feeding decisions driven only by caregivers. The present study investigated conceptualizations related to the CF period among caregivers living in the United States and how they influence, and are influenced by their young children. Rather than focusing solely on what foods were offered and when, this framework highlights how feeding practices are fostered from the interaction of caregiver beliefs and values, physical and social settings, and children’s developmental characteristics. Applying the Developmental Niche framework, we identified environmental influences at the household level and how they shape children’s opportunities for mastery and learning in the eating domain [17]. Caregiver practices shaped the child’s experiences with food and eating, ultimately, through interactions during family mealtimes, acquainting children with parental eating values and the culture and ideals of the extended family and larger society. Children also bring their individual personalities and preferences to the family eating realm, thus influencing family dietary choices and practices.

Caregivers’ feeding decisions reflected broader cultural values emphasizing health optimization, safety, and the development of autonomy, consistent with Western feeding norms and, to some extent, best practice guidelines promoted by health care professionals [1,31]. While complementary feeding practices vary widely across cultural contexts, caregivers in this study described shared goals of supporting children’s nutrition and development and fostering autonomy and self-feeding skills while introducing them to culturally relevant family foods and mealtime routines.

Interpreted through the Developmental Niche, caregivers’ beliefs about complementary feeding functioned as ethnotheories that organized decision-making under conditions of uncertainty. Appropriate first foods were deemed to be plant-based and most often fruit, vegetable, and/or infant cereals, aligning with best practice guidelines aimed at increasing children’s intake of healthful foods and avoiding early introduction of sugars to prevent the development of preferences for sweet foods [32,33,34,35]. Caregiver practices also related to reports of contamination of infant cereals [36] which have heightened consumer concerns about their safety [37]. Caregivers actively interpreted information about nutrition, safety, and development in light of prior experiences, cultural messaging, and desired long-term outcomes for their children’s eating. At times, this information was attributed to advice from their primary care providers and other times from sources that were less evidence-based (i.e., blogs, social media, etc.). Rather than passively adopting guidance, caregivers adapted recommendations to fit their values, perceptions of child readiness, and family circumstances, underscoring that CF decisions are individualized for the child and family.

The majority followed current guidance [31,38,39] and offered first foods around 6 months of age, although some waited as long as 12 months or longer, particularly in the case of animal source foods. Most research has focused on *early* introduction of solid foods and the associated health risks [40,41]. However, fewer studies have been reported or focused upon the effects of introduction to solids *after* the suggested timing of 6 months, despite the cautionary messages by professional organizations regarding the potential negative developmental impacts upon children’s eating and food preferences if the critical window for solid food introduction is passed [31,42]. Moreover, delaying the introduction of micronutrient rich foods (i.e., bioavailable iron- and zinc-rich foods), particularly for the human milk-fed infant, confers risk for iron and zinc insufficiency—micronutrients which are critical for optimal growth and development [43]. Caregivers’ beliefs about timing, texture, and food selection were closely tied to perceptions of child readiness and safety, illustrating how ethnotheories about development can shape CF feeding decisions.

Food parenting during CF was described as an intentional process of supporting children’s learning and autonomy while balancing competing family and work demands. Caregivers emphasized responsiveness, flexibility, and patience as children developed eating skills, acknowledging that ideal feeding practices were sometimes difficult to sustain amid time constraints, convenience needs, and family responsibilities. Primary values and beliefs focused on nutrition adequacy and safety but also with the goal of helping the child foster a healthy relationship with food and eating. These findings align with prior work, highlighting the tension caregivers experience between feeding ideals and the realities of family life. In a recent qualitative systematic review, Dattilo and colleagues (2020) report that parents must consider cost, convenience, and access to family/traditional foods when selecting both the foods offered and the method of feeding their young children [44]. The dissonance of striving to meet health and feeding ideals vs. the demands of family life and the whimsical nature of young children’s food preferences continue into the preschool age, as also noted by others [45,46].

Caregivers expressed a range of emotions about CF experiences, from concern, angst, and uncertainty to happiness and joy. Food parenting during CF was described as being “uncharted territory” that required “learning as you go.” Previous qualitative reports have emphasized the frustration and difficulty of feeding, the anxiety experienced if the child is not eating well [46], and parents seeking emotional support and comfort during challenging feeding times [47]. However, our participants also spoke of the joy of watching children learn to master eating skills and their transition to foods of the family table.

Consistent with the Developmental Niche framework, children’s developmental characteristics played an integral role in shaping caregiver feeding decisions. Caregivers described closely attending to this particular children’s motor abilities, preferences, and communicative cues, adapting feeding practices as this particular child’s skills and interests evolved. If practices used with previous children were not effective, then flexibility was required for the current child. These caregiver thoughts and practices echo what has previously been reported: though children in the same family share genes, there are considerable differences in their traits and behaviors, including appetitive traits [48]. Therefore, as noted long ago by Dunn and colleagues [49], siblings behave differently because, even though they share a household and caregivers, food, feeding practices, and feeding environments cannot be assumed to remain the same for different children at different times. These findings underscore CF as a bidirectional learning process, in which children actively co-construct their feeding environments through their responses and behaviors.

Caregivers viewed mastery of eating as emerging through repeated interactions, trial-and-error, and practice within everyday routines. While approaches varied, caregivers commonly described adapting strategies in response to children’s autonomy-seeking behaviors and emerging competencies. Later in the CF period, children’s expressive language begins to emerge and receptive language related to food and eating also increases [50,51] and dovetails with expressions of liking and food rejection. In our sample, the response to children’s increasing communication was largely positive—caregivers enjoyed being able to interact with their children and to better understand their needs and preferences. From many caregivers’ perspectives, these signs of increased maturation gave insight into their children’s developing personalities and the process of “becoming their own person,” highlighting the dynamic nature of caregiver–child interactions during CF.

Family mealtimes served as important contexts for learning and socialization, providing opportunities for modeling, imitation, and shared engagement around food. Caregivers described these interactions as meaningful indicators of children’s readiness to participate more fully in family eating practices. Harrist and Waugh (2002) describe these types of transactions as examples of “dyadic synchrony”: a type of interaction between the caregiver and child that is mutual, reciprocal and harmonious [52]. They argue that children’s learning from these kinds of episodes can support their development (or in some cases of over-controlling interactions, inhibit it).

The integration of the child into family mealtimes emerged as a central goal across caregivers in this study. Eating together was valued for fostering togetherness, simplifying meal preparation, supporting children’s social and eating development, and bringing caregivers a sense of joy. Family mealtimes yield many positive outcomes for children and families including positive associations with diet quality as well as psychosocial outcomes like academic performance and social competence [53]. Viewed through the Developmental Niche framework, family meals represent a key setting and opportunity in which caregiver beliefs, contextual considerations, and child competencies converge to shape eating development across the lifecourse.

## 5. Limitations

This study has several limitations that should be considered. This study followed qualitative guidelines for sample size [54]; however, the characteristics of this sample preclude us from generalizing to other groups in the US or globally. The sample was relatively homogeneous, consisting primarily of educated mothers from partnered households, and does not reflect the diversity of CF beliefs and practices across cultural, socioeconomic, and global contexts. Methodologically, we opted to collect sociodemographic data, including information about food security, at the outset of the interviews. We did this to ensure that we could characterize our sample; however, it is possible that this created social desirability biases in interviewees’ responses. This could have been the case for those who responded that they experienced a level of food insecurity (5 participants reported a level of food insecurity by answering “sometimes true” to our questions). Data collection occurred during the COVID-19 pandemic, which may have influenced caregiver experiences, although this was not explicitly noted by participants.

Moreover, perspectives were limited to primary caregivers of typically developing children, and findings may not generalize to families of children with disabilities or growth concerns. Our decision to include only families with typically developing children should not minimize the importance and differences that likely would have emerged if families with children with disabilities had been included. We strongly endorse that additional research should be undertaken to highlight the beliefs, practices, experiences, and constraints for CF for these families with special needs and considerations. In addition, we elected to focus on primary caregivers as a starting point, rather than other caregivers (e.g., grandparents, other child care providers, and health care professionals). Future research should include other influential caregivers and information sources as they are important influencers of caregiver decisions.

Lastly, our team was composed of experienced researchers, many of whom have previous experience conducting qualitative research. However, it must be acknowledged that all were female and White, which may have influenced both participant responses as well as the interpretation of their responses.

## 6. Conclusions and Implications

Applying the Developmental Niche framework offers a novel, theory-driven lens for understanding CF as a dynamic, contextual, and reciprocal process. First, caregivers choose food and feeding methods that they believe support adequate nutrient ingestion for children’s health and development, and that may or may not be in alignment with existing CF guidelines. Caregivers seek to build a repertoire of food acceptance that meshes with preferred family foods but also consider their child’s likes and dislikes. Responsively and joyfully teaching their child to master eating skills, while supporting children’s increasing autonomy, is a central value. Caregivers aim to help their child build a long-term healthy relationship with food and eating. Lastly, caregivers seek to enculture their child to family foodways and traditions of the surrounding culture.

The challenge ahead lies in building a stronger evidence base to support caregivers in achieving their feeding goals. The CF period is unquestionably a critical period of growth and brain development, yet the 2025 Dietary Guidelines for Americans Committee Scientific Report concludes that there is insufficient data for nearly all of the CF-related questions addressed for this age group [55]. This lack of conclusive data spans key areas—including optimal timing of introduction of solid foods, associations with growth and development, and the long-term impacts of early food choices and feeding practices during the CF period on risk for chronic disease and health. To address these gaps, rigorous longitudinal studies of young children’s eating behaviors and caregiver feeding practices are urgently needed—including those grounded in conceptual frameworks such as the Developmental Niche—which are designed to capture the dynamic, bidirectional nature of caregiver–child interactions that shape the development of healthful eating. Such studies should focus on families from diverse cultural backgrounds and on families with children with special needs. Engaging caregivers, by developing messaging that aligns both with existing guidance and with their cultural traditions, continues to be an important challenge for nutrition educators and other health care professionals. Frameworks such as the Developmental Niche may be particularly valuable for advancing evidence-based guidance that reflects the realities of family life and the bidirectional nature of early eating development.

## Figures and Tables

**Table 1:** Interview guide for caregivers on complementary feeding of infants from the *First Foods* Study.

Thank you so much for making time to meet with me. Today we will be talking about introducing new foods, and especially meat, to your child. Talking about feeding decisions can be a sensitive topic for some parents. I will ask a series of questions, and you may choose not to answer any question. You know your child better than anyone, and I am here to learn from you, not to judge.
Before we begin, I would like to verify your information:
- How old is your baby/toddler? - What sex is your baby/toddler? - How many children are in your household? - What race or ethnicity do you identify as? - What gender do you identify as?
Thank you for verifying that for me. Do you have any questions for me before we begin?
To start, I have some questions about how you decided to introduce new foods to your child.
1. What was the first food you offered to your child? How old was your child when you introduced this? What form was that food in? Pureed, mixed with breast milk or formula, solid but soft, cut up into small pieces? Why was that food important to introduce first? Was there anything about that specific food that was important for your child? 2. In what order did you offer different food groups, like fruit, vegetables, dairy, and meat? How did you decide this? What information informed your decision? Probe: where did that information come from? Can you describe how you introduced these foods to your child? Probe: Did you feed them? As a finger food? Off of your plate? Probe: Was this food prepared specifically for them? Did you prepare it, or did you buy it prepared? What do you like about feeding your child solid foods? What is going well? 3. Are there any foods you have avoided introducing? If so, why? Do you intend to offer these foods eventually? 4. If the parent has other children: Did you do anything differently when introducing solid foods to this child? Probe: if so, what was different? If so, how come you did things differently? 5. What was the first animal-source food you offered your child? For example, eggs, meat, fish, yogurt? 6. How old was your child when you first offered them this food? 7. If eggs or dairy were introduced first, how old was your child when you offered them meat? What was the meat? How come you decided to introduce meat to your child at that time? 8. If eggs/dairy and meat were offered at different ages: why were eggs/dairy and meat offered at different ages? 9. How do you respond if your child did not want to eat other foods on the plate? 10. How do you plan to help your child transition to eating the same foods the rest of your family eats?

**Table 2:** Participant Characteristics of caregivers participating in the *First Foods* qualitative study (N = 46).

Caregiver Characteristics	N (%)		Child Characteristics	N (%)
Women Caregivers	38 (83)		Child Age	
Caregiver Race/Ethnicity			6–11 Months	19 (41)
Asian American	1 (2)		12–17 Months	15 (33)
Black	10 (22)		18–24 Months	12 (26)
Hispanic	3 (7)		Female Children	24 (52)
Hispanic, White	1 (2)		Child Race/Ethnicity	
Native Hawaiian/Pacific Islander	1 (2)		Asian American	1 (2)
White	30 (65)		Asian American, White	1 (2)
Marital Status			Black	10 (22)
Single	2 (4)		Black, Hispanic	2 (4)
Married/Partnered	41 (89)		Black, White	2 (4)
Separated/Divorced	3 (7)		Hispanic	1 (2)
Education			Hispanic, White	2 (4)
Some High School	1 (2)		Native Hawaiian/Pacific Islander	1 (2)
High School	1 (2)		Native Hawaiian/Pacific Islander, White	1 (2)
Some College	5 (11)		White	25 (54)
Vocational Training	2 (4)		Age at Complementary Food Introduction	
Associate’s Degree	4 (9)		<4 months	4 (9)
Bachelor’s Degree	19 (41)		4–6 months	27 (57)
Graduate/Professional	14 (30)		>6 months	7 (15)
Employment			Not reported	10 (22)
Employed Full-time	22 (48)			
Employed Part-time	12 (26)			
Not employed	11 (24)			
Student	1 (2)			
Single-child Household	8 (17)			
Household with low income *	13 (28)			
Food-insecure Household	11 (24)			

* Below the Federal Poverty Household Limit [24].

**Table 3:** Themes from the qualitative interviews about complementary feeding conducted with caregivers of infants from the *First Foods* study.

Theme/Subtheme	Quotes
**Caregivers’ Approach to Introducing Solid Foods with Both Anticipation and Concern** Timing and order of CF offered to children Foods caregivers avoid offering	But I got some more baby purees with the fruits, the vegetables, um, and eased into it I think chicken just because it’s the mildest. Um, so I felt like the fruits might give him a good start, you know, a good taste. And maybe he would be more open to the less tasty foods from there, yeah. But like um, yeah, I think just the way that we’ve kind of introduced foods to her and the way her tastes have developed maybe because of that um, that’s kind of fun. We finally had allergy testing like this week and so she was cleared on peanuts so I’ve been like crazy cautious with peanuts so I just kind of introduced peanut butter like this past week for her I gave her like a lick. Um so I was avoiding that for a little bit. You know, obviously, like, “watch for the choking hazards.” You know, I think that choking hazards is one of the biggest things… I’ve tried to stay away from as much like really, really, really artificial, like you know, sweet stuff and things like that for now. She hasn’t really tried too too much of like sweets, you know, I don’t really want her to get started too young on that kind of thing, you know, I just—so if she has very few teeth, I don’t want to write them all out yet. No, they told us that he was ready for meat, um, way before we had offered it. We started offering it we, we held we held out a little bit longer. Um, I think I think more than anything it was because of the digestive issues. I felt like his digestion was a little slower, you know how they kind of grow into it… …We graduated to the meats. I’m going to say he was probably about nine months because he had his, he had his front teeth. I wanted to make sure he had some teeth.
**Caregivers’ and children’s learning** Children’s rapid learning and skill development Concurrent rapid demands for changes in food parenting	Um, he would push it out with his tongue and just crinkle up his face. Just did not want it. You could tell, you could tell he wasn’t big on it. He definitely was just kind of like, “This tastes good,” like you could see the wheels turning like, “This tastes good, it’s hard, I have to do something with it, but I’m not sure what to do with it yet.” So he literally just like, sat there with it in his mouth for like, a whole minute before he actually did anything with it. He was just kind of sitting there and you could just see it processing of like, “My tongue can’t crush it, maybe I need to use my teeth.” Like, It was a very odd process to watch him go through, but it was kind of fun like, because you could just see the thoughts going through his head. It was really cool. Also, I enjoy seeing her eat. She’s so cute when she eats. She-- she gets so excited and she always eats everything there is, she never leaves anything behind. Yeah, it’s cute. It’s a-- it’s a bonding moment too. It was early on. Yeah it was like when she was learning the pincher grasp. So I think like I would give her a little bit in her mouth and then she would kind of play with it. Um but yeah it was small where she could pick it up. How to eat– so using utensils, how to manipulate the food and get it to their mouth, which sounds like a basic concept, but it’s- it’s- it’s a lot of process involved with, like, “pick up food,” “put in mouth,” “figure out how to chew.” Uh, sensory, so being exposed to the yogurt, which is gushy, the quinoa, which might be pebbly and have like a different texture than beef is, maybe it takes a little bit more jaw movement. Um, but I think that the things that you have to gnaw on are also important too, for jaw development and their- their actual physical palette. So those types of- of skills that are really kind of shaping how they think. I think what’s important too is, like, a relationship—what kind of relationship you have with food. Um, I guess not seeing like any food as bad. You know, see like, you know, every food is helpful in some regard, like some capacity and not to feel like there’s any forbidden food or feel bad about it. And just being able to listen to her body. I guess it’s the biggest one that, you know, you’re listening to your stomach, if you’re hungry, you need to eat and if you’re full, that’s fine, you’re full. Like just listen to those cues. It’s rewarding to see them try like different things and like even not just like flavor wise like I let my kids make a mess because I know it’s important to feel different things like different textures and their fingers and stuff like that. So, I just think that it’s really cool to watch them like explore and learn. Yeah, everything’s new, and I mean, that’s good, that’s that’s the exciting part of this chapter in life. I just offer them a little bit and you know, they say like I tried to do that whole 10 times thing, offer it to them at least 10 times. And um, if, if they still don’t eat it after that. Okay, wait a little bit, you know, before you introduce it again, but I realized that you know, his tastes are different than mine, you know, I’m not going to give them just what I like. I want him to, to be open to different things. She went through, and all of my kids have done this, gone through a phase of picky eating, like not eating much, not eating anything, refusing things that I know they enjoy or they enjoyed once before, and I kind of came to really not sweat it by number five and also just to chalk it up to teething, it could have been something else. But we always really did like persevere and kind of push through. We never give in to—like we always offered those things… you didn’t have to eat them, but I wasn’t going to make something else specific. At first he did not like them but I kept trying it and now he, now he likes them. So I think he was just not used to it at first. Right around the six month mark he was really like, I need food and I need food right now and I was like okay, okay, and that really helped, helped me and him I’m assuming as well transition from like okay we’re eating solid foods and now we’re napping and how does this correlate? I’ve been more focused on her gross motor development and her communication development. I would maybe cut it into very small cubes or something and put it on the tray in front of her and let her start to pick it up and feed it to herself as a finger food. Um, and then the more teeth she gets, then I will branch out into things that it might be harder to chew. But that, I think, has probably been one of the bigger obstacles is just with us both back at work full time, and I’m also in the office more, like, it was really easy in the beginning; I was working from home full-time. I also thought it was just like really convenient because you can just feed them off your plate. You don’t have to be carrying around the jars, like the convenience factor, but huge into like the cultural thing. I just got tired of, uh, doing stuff separately, so I was like okay, you know, she’s just gonna eat some of our food slowly.
**Drivers of Caregivers’ Decisions Related to Offering Solid Foods to Their Children**	I want him to get his carbs, his vegetables, his fruits and you know, his grains, um his protein, um you know, the, the calcium. Protein is a huge thing, and I’m starting to learn a lot more about like, what protein can do for your body. I just learned recently from my doctor that a lot of foods that we think has a lot of fiber doesn’t. So, I’ve started looking at the labels. She liked it. She likes all foods. Um just gobbling it up, reaching for the spoon, wanting, indicating more basically. So, a lot of it is texture. Some of it is also that WIC gives you an incredible amount of eggs. We were on WIC and we had a lot of baby food, it’s what I had. And that’s, I know that sounds so lazy, but like it’s, there are some that I can totally stick in the microwave. Um, and there’s some like, okay… like pot pies and stuff…I mean they’re laden with sodium, but they’re pretty easy… But like when we get home from being out and having appointments and stuff, like I’ve got people to feed them fast. Um, and so if, you know, I guess it’s just a matter, it’s a matter of convenience. And then the vegetable worry is more about that they’re used to be more vegetables he would eat. And he has really been narrowing what vegetables he will even put near his mouth and he doesn’t like crunchy things…Um, and he doesn’t like like mushy things… We’re trying to gradually build up to, uh, foods with more texture… thicker… I kind of just mix the meat and the vegetable because I know that sometimes the meat has like a harsh taste and sometimes they don’t like it or they don’t really like the texture of it. We kind of came, well at least I came from the clean your plate club. “Clean your plate!” “Clean everything off your plate!” And I think that has become more… realizing that that’s sort of a damaging way of raising kids, that it’s okay if you’re not hungry; you can stop.
**The Goal of CF: Integration of the Child into Family Mealtimes**	Because that’s what we eat, that’s how we enjoy it. I want [him] pretty much eating everything that we eat by the time he’s one. Um, but I just make, we try to make sure it’s a family thing. We try to sit down all together and eat. So slowly she started eating up. She wouldn’t eat much, but at least the fact she was eating made me happy. So, I use the process as a means of transmitting her to learn more about what the family love to eat, and also, um, cultural or family meal. For example, in my family, we love to eat food a lot, especially watermelon, watermelon. We love to eat it a lot, a lot, almost daily. So, uh, seeing us eating it with other family members, that’s like teaching her this is what our family love to eat. So, you too have to get used to it.

## Data Availability

The raw data presented in this article are not readily available because they are video-recordings that would de-identify the participants. Requests to access the de-identified transcripts should be directed to the corresponding author.
